# Depression and anxiety disorder in hyperemesis gravidarum: A prospective case-control study

**DOI:** 10.4274/tjod.78477

**Published:** 2017-12-30

**Authors:** Yusuf Topalahmetoğlu, Mehmet Metin Altay, Derya Akdağ Cırık, Yusuf Aytaç Tohma, Eser Çolak, Bora Çoşkun, Orhan Gelişen

**Affiliations:** 1 University of Health Sciences, Etlik Zübeyde Hanım Women’s Health Training and Research Hospital, Clinic of Obstetrics and Gynecology, Ankara, Turkey

**Keywords:** Hyperemesis gravidarum, pregnancy, depression, anxiety disorder

## Abstract

**Objective:**

To assess the anxiety and depression status of women with hyperemesis gravidarum (HG); the risk factors for developing both depression and anxiety in women with HG were evaluated.

**Materials and Methods:**

A total of 200 women, 100 diagnosed as having HG before the 20th week of gestation at a tertiary referral center and 100 gestational-age-matched controls were enrolled. The socio-demographic data and the depression and anxiety scores, as assessed using the Beck depression and anxiety inventory were compared between the two groups.

**Results:**

The median depression and anxiety scores were significantly higher in the HG group compared with controls (19.5 vs. 9.0 and 22.0 vs. 10.0). Women with HG have the highest relative risks for moderate depression and severe anxiety [relative risk (RR): 16.88 and RR: 20.50, respectively]. In the univariate analysis, having HG, low education level, low income and poor social relationships were significant predictors of depression and having HG. Moreover, poor social relationships significantly predicted the presence of anxiety disorder. However, having HG and poor social relationships were found as the only independent predictors of both depression and anxiety. Patients with HG were 5.5 and 6.7 times more prone to having depression and anxiety disorder compared with controls, respectively.

**Conclusion:**

Both depression and anxiety disorder were more frequent in women with HG who have weak family and social relationships, lower education and income levels. Therefore, the determination of the psychological status of women with HG should be an integral part of the evaluation.

## PRECIS:

We showed that having weak family and social relationships, lower socio-economic status played an important role in the development of depression and anxiety in pregnant women.

## INTRODUCTION

Nausea and vomiting during early pregnancy is very common and generally accepted as a part of normal physiology^(1,2)^. Hyperemesis gravidarum (HG) is a pregnancy condition characterized by severe nausea and vomiting starting before the 22^nd^ week of gestation. Although it generally ends before the 16^th^ week, HG may be severe in 2% of pregnant women, who require hospitalization^(1,2)^. Although there is no universally accepted criteria for the diagnosis, HG is characterized by persistent vomiting and nausea, weight loss of more than 5% of pre-pregnancy body weight, ketonuria, electrolyte abnormalities (hypokalemia), and dehydration (high urine specific gravity), resulting in the diminishment of the woman’s quality of life and a significant contribution to health care costs and time lost from work due to persistent vomiting^(3)^. Despite its frequency, the etiopathogenesis of HG has not yet been clearly elucidated. Many theories based on endocrinologic and metabolic factors, gastrointestinal dysfunction, and immunologic, genetic, and psychological factors have been proposed^(2,3,4)^. Besides its physical symptoms such as dehydration and electrolyte imbalance, HG can also affect quality of life and the psychological state of pregnant women^(5,6)^.

Depression is the most common psychological disease seen in women, and encountered in 14% to 48% of pregnant women^(7,8,9,10)^. Although recent studies demonstrated higher rates of depression and anxiety in pregnant women with HG, few studies have evaluated the predictive factors or cause-and-effect relation of these psychological disorders and pregnancy^(11,12,13,14,15)^.

In this case-control study, the anxiety and depression disorder of pregnant women with HG was assessed, and we aimed to determine the risk factors for developing both depression and anxiety in pregnant women.

## MATERIALS AND METHODS

The study was conducted in a tertiary care center, Etlik Zübeyde Hanım Women’s Health Training and Research Hospital from June 2013 to October 2013. The local ethics committee approved the study (approval number: 2013-165) and all participants gave written informed consent. The trial was performed in accordance with the Declaration of Helsinki. Pregnant women diagnosed as having HG before the 20^th^ week of the current viable pregnancy and required hospitalization for intravenous fluid replacement were included in the study group. Persistent vomiting accompanied by weight loss exceeding 5% of pre-pregnancy body weight, an objective measure of acute starvation (usually large ketonuria on urine analysis), electrolyte abnormalities and acid-base disturbances, was diagnosed as HG. Gestational-age-matched controls were recruited from patients who came for routine antenatal care follow-ups. Patients who had multiple pregnancies, thyroid disease, prior psychiatric disease or conditions with elevated serum human chorionic gonadotropin levels such as gestational trophoblastic diseases and chromosomally abnormal fetus were excluded.

All participants were asked to complete a demographic and socioeconomic data collection form. In this form, patients assessed and rated their relations with other family members and society as strong or weak.

### Anxiety and depression scores

The status of depression and anxiety was evaluated using the Beck Depression Inventory (BDI)-II and Beck Anxiety Inventory (BAI). The BDI includes a 21-question self-assessment scale and answers were scored from 0 to 3. BDI scores were grouped as follows: 0-9 as no depression, 10-16 as mild depression, 17-23 as moderate depression, and 24-63 as severe depression. Answers to each question in the BAI were scored from 0 to 3. The BAI scores were classified as follows: 0-7 as no anxiety, 8-15 as mild anxiety, 16-25 as moderate anxiety, and 24-63 as severe anxiety.

### Statistical Analysis

Statistical analysis was performed using SPSS 21.0 (IBM Corp. Released 2012. IBM SPSS Statistics for Windows, Version 21.0. Armonk, NY). Univariate analyses to identify variables associated with anxiety and depression were investigated using appropriate statistical tests such as Student’s t-test, the chi-square test and Mann-Whitney U tests. The Kruskal-Wallis test was used to compare the anxiety and depression scores between the two groups in terms of different variables. Bonferroni correction was used to test the significance with pairwise differences to adjust multiple comparisons. The association between ordinal variables was investigated, and correlation significance was calculated using the Spearman test. For multivariate analyses, possible factors identified were further entered into the logistic regression analysis to determine the independent predictors of anxiety and depression. A 5% type 1 error was used for the statistical significance.

## RESULTS

A total of 100 pregnant women with HG and 100 gestational-age-matched controls were included in the study. The socio-demographic data of all participants are listed in Table 1.

Using BDI, the median depression scores were significantly higher in the HG group compared with controls (19.5 vs. 9.0; p<0.001). Similarly, using BAI, the median anxiety scores were also higher in the HG group compared with controls (22.0 vs. 10.0; p<0.001). The percentage of pregnant women living on minimum wage was nearly double in the HG group compared with controls (61.9% vs. 38.0%; p=0.01). The education level was also significantly lower in the HG group compared with controls such that the percentage of women who graduated from at least high school among the HG and control groups were 40.4% and 59.6%, respectively (p=0.005). Pregnant women who defined their relationship with their families as “weak” had a higher percentage of HG than those who defined them as “strong” (69% vs. 31.0%; p=0.027). Similarly, women who had weak social relations were also more prone to having HG (63.6% vs. 36.4% p=0.041).

When all other possible contributing factors were matched, patients in the HG group had a higher risk of having depression or anxiety (mild, moderate or severe) compared with controls (Table 2a, 2b). The relative risk of women with HG was highest for moderate depression and severe anxiety (16.88 vs. 20.50).

Data of all participants were analyzed to determine the risk factors for depression and anxiety. HG, lower educational status, lower income, and poor social relationships were the significant risk factors for depression and HG, and poor social relationships was a significant risk factor for anxiety in the entire pregnant population (Table 3). Additionally, HG and poor social relationships were independent risk factors for both depression and anxiety in the entire cohort. Patients with HG were 5.5 times more prone to having depression and 6.7 times more prone to having anxiety compared with the matched controls. Additionally, risks of having depression and anxiety in pregnant women with poor social relationships were 2.8 and 5.6 times higher, respectively, when compared with controls (Table 4).

## DISCUSSION

The psychological theory for describing the pathogenesis of HG suggests that either the presence of conversion or somatization disorder, or the exaggerated response of a patient to stress may cause HG^(16)^. Although nausea and vomiting during pregnancy were more commonly seen in dependent, hysterical, depressive, and anxious women, severe and persistent vomiting itself might also cause the psychological problems in patients^(17,18,19,20,21,22)^.

In this study, we investigated the possible effects of many socio-demographic parameters on HG. Among these parameters, some (age, parity, previous miscarriage, age of marriage and working status) were found not to be related, whereas others (income level, education level, social and family relations) were found related. Anxiety and depression were significantly more common and more severe in the HG group than in the controls. Similar to Hizli et al.^(15)^, we also found the incidence of depression as 84% in the HG group. This high incidence of depression might be attributed to the completion of questionnaires on the first day of hospitalization and high percentage of patients with low income. In the univariate analysis, patients with low income, low educational status, and poor social relationships had increased risk for having depression in the current study. An alternative view is that HG is an independent variable that increases the relative risk of all stages of anxiety and depression. The most dramatic effect of HG was seen on the development of severe anxiety. A woman with HG was 20.5 times more prone to having severe anxiety compared with healthy pregnant woman in the present study. These findings are consistent with some previous studies; however; some other studies could not demonstrate an association between severity of HG and depression^(19,20,21)^.

Predictive factors for developing anxiety disorder and depression in pregnant women with HG were also investigated in a few studies^(11,14,17)^. Tan et al.^(14)^ investigated the incidence and risk factors for developing anxiety and depression in a cohort of 209 women with HG in early pregnancy. HG was found to be the only independent risk factor for developing anxiety, and the only independent protective factor for developing depression was the history of miscarriage. In 2012, Hizli et al.^(15)^ evaluated the impact of HG and socio-demographic variables on depression in Turkish patients during pregnancy. They used the BDI-II and reported the incidence of depression as 80% in the HG group and 5% in the control group. They also stated that the presence of HG was the most important predictor of depression, and additionally, young maternal age and poor family interaction were other weaker contributors. Recently, Annagür et al.^(11)^ investigated the association between HG and eating attitudes on anxiety and depression symptoms in 48 women with HG and 44 controls. The authors concluded that HG was associated with symptoms of anxiety and depression but not with eating attitudes.

### Study Limitations

The strengths of the study are the use of objective inclusion criteria and a relatively high number of patients, and the limitations are the use of psychiatrics test for depression instead of examinations by a psychiatrist, and the matching process between the study and control group was insufficient.

## CONCLUSION

In this study, we showed that both depression and anxiety disorders were more common and severe in patients with HG compared with controls. In addition, having weak family and social relationships and lower socioeconomic status also played an important role in developing depression and anxiety in pregnant women. Therefore, the psychological state of patients should also be noticed during the evaluation for physical health of women with HG.

## Figures and Tables

**Table 1:**
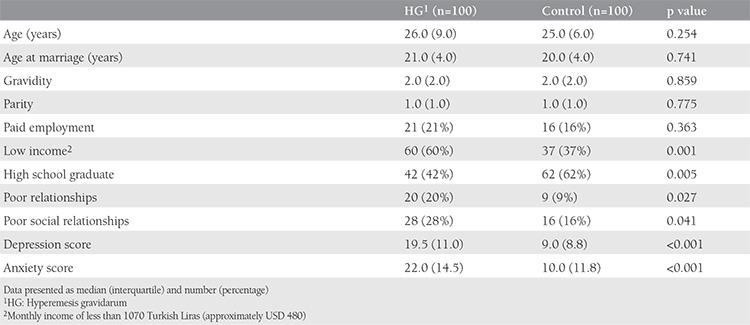
Socio-demographic characteristics of the hyperemesis and control group

**Table 2a:**
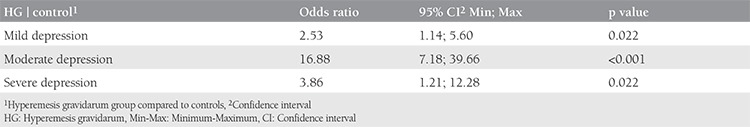
The relative risk of depression (a) or anxiety (b) in the hyperemesis and control groups

**Table 2b:**
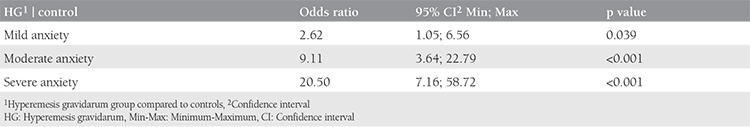
The relative risk of anxiety (b) in the hyperemesis and control groups

**Table 3:**
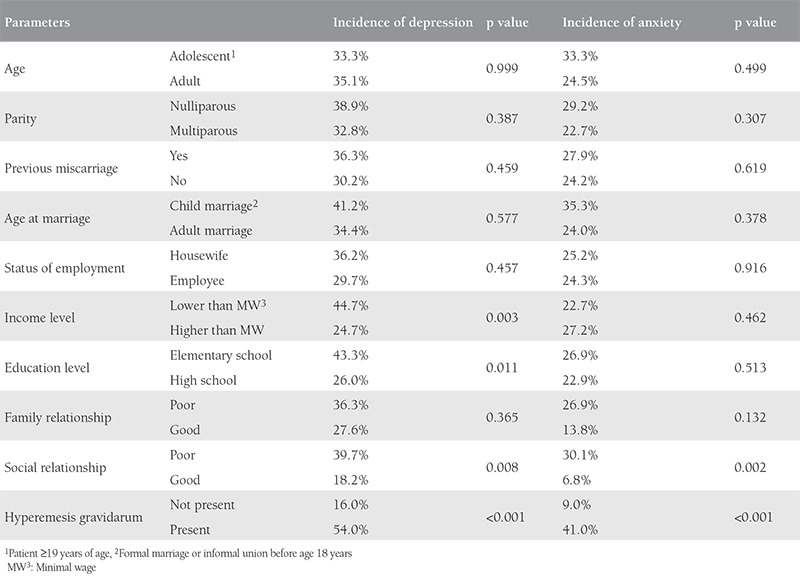
Parameters tested to be risk factors for depression or anxiety in early pregnancy: univariate analysis

**Table 4:**
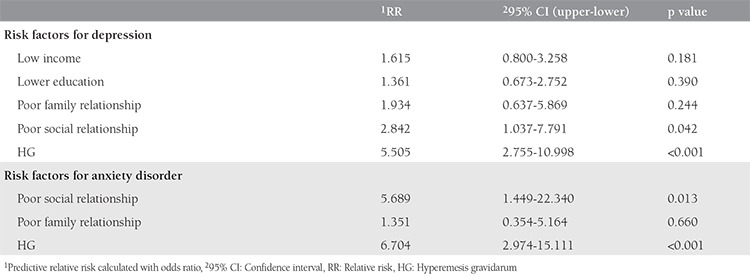
Relative risk ratios of various parameters for depression and anxiety: multivariate analysis
